# Is there an association between elevated or low serum levels of phosphorus, parathyroid hormone, and calcium and mortality in patients with end stage renal disease? A meta-analysis

**DOI:** 10.1186/1471-2369-14-88

**Published:** 2013-04-17

**Authors:** Jaime L Natoli, Rob Boer, Brian H Nathanson, Ross M Miller, Silvia Chiroli, William G Goodman, Vasily Belozeroff

**Affiliations:** 1 Cerner Research, Culver City, CA, USA; 2 OptiStatim, LLC, Longmeadow, MA, USA; 3 Amgen Europe, Zug, Switzerland; 4 Amgen, Thousand Oaks, CA, USA

**Keywords:** Calcium, Dialysis, Meta-analysis, Mineral metabolism, Mortality, Parathyroid hormone

## Abstract

**Background:**

Biochemical markers of altered mineral metabolism have been associated with increased mortality in end stage renal disease patients. Several studies have demonstrated non-linear (U-shaped or J-shaped) associations between these minerals and mortality, though many researchers have assumed linear relationships in their statistical modeling. This analysis synthesizes the non-linear relationships across studies.

**Methods:**

We updated a prior systematic review through 2010. Studies included adults receiving dialysis and reported categorical data for calcium, phosphorus, and/or parathyroid hormone (PTH) together with all-cause mortality. We performed 2 separate meta-analyses to compare higher-than-referent levels vs referent and lower-than-referent levels vs referent levels.

**Results:**

A literature review showed that when a linear relationship between the minerals and mortality was assumed, the estimated associations were more likely to be smaller or non-significant compared to non-linear models. In the meta-analyses, higher-than-referent levels of phosphorus (4 studies, RR = 1.20, 95% CI = 1.15-1.25), calcium (3 studies, RR = 1.10, 95% CI = 1.05-1.14), and PTH (5 studies, RR = 1.11, 95% CI = 1.07-1.16) were significantly associated with increased mortality. Although no significant associations between relatively low phosphorus or PTH and mortality were observed, a protective effect was observed for lower-than-referent calcium (RR = 0.86, 95% CI = 0.83-0.89).

**Conclusions:**

Higher-than-referent levels of PTH, calcium, and phosphorus in dialysis patients were associated with increased mortality risk in a selection of observational studies suitable for meta-analysis of non-linear relationships. Findings were less consistent for lower-than-referent values. Future analyses should incorporate the non-linear relationships between the minerals and mortality to obtain accurate effect estimates.

## Background

Among patients with end stage renal disease (ESRD), the deterioration of kidney function is often accompanied by secondary hyperparathyroidism (SHPT) involving disturbances in mineral metabolism, including elevated levels of serum phosphorus, decreased calcium, and elevated parathyroid hormone (PTH) [1,2]. Observational studies suggest that biochemical markers of altered mineral metabolism are associated with poor clinical outcomes among patients with ESRD requiring dialysis, although controversy exists regarding the strength of the evidence and the independent nature of these relationships [3-16].

In a systematic review published in 2008, Covic et al. [17] identified 22 studies published from 1980–2007 that assessed the association between disturbances in biochemical parameters and all-cause mortality among patients with chronic kidney disease (CKD), including 19 studies of patients on dialysis. The authors noted that the studies were too clinically and methodologically diverse to permit an appropriate meta-analysis. Nevertheless, a qualitative assessment of the studies led them to conclude that elevated values in certain biochemical parameters, specifically the serum levels of PTH, calcium, and phosphorus, as well as very low values of phosphorus, were associated with an increase in mortality risk among patients on dialysis.

In contrast to Covic et al., Palmer et al. [18] concluded from their meta-analysis that calcium and PTH did not have statistically significant associations with mortality, but an association between high serum phosphorus and mortality was observed (relative risk [RR] = 1.18, 95% confidence interval [CI] = 1.12-1.25). The Palmer et al. meta-analysis assumed a linear relationship between the serum levels of the 3 biochemical parameters and all-cause mortality, a limitation that they acknowledged could have biased their results.

**Figure 1:**
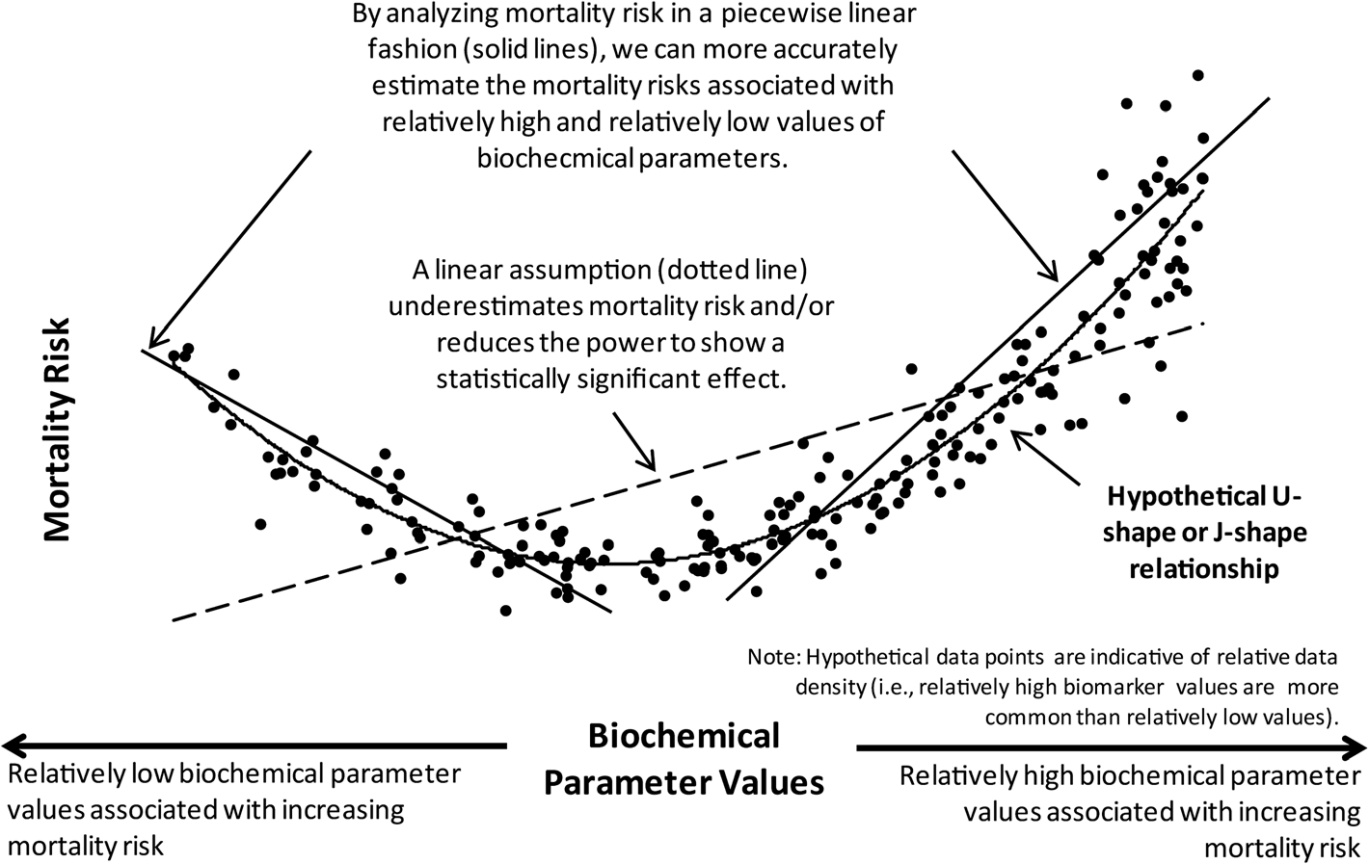
Hypothesized relationship between biochemical parameters and mortality risk.

How the biochemical parameters are modeled is crucial. If the true relationship between the biochemical parameters and mortality is indeed U-shaped or J-shaped, then imposing linear assumptions on a model (either within an individual study or in a meta-analysis) would not accurately quantify the true association (see Figure 1). Often a continuous risk predictor such as age is modeled “as is” without transformations. By doing so, a linear relationship between the predictor and outcome is assumed. If its risk ratio is significantly above 1, then the model implies that for every additional unit (eg, year), the risk increases by a certain constant amount. However, it is possible that both very high and very low values of a variable could increase the risk. Some studies have demonstrated non-linear associations (eg, U-shaped, J-shaped) between phosphorus, calcium, and PTH and mortality risk whereby both low and high serum levels of these variables are associated with an increased risk of death [10,14-16,19-24]. In addition, there is evidence suggesting mortality risk is influenced by interactions between serum calcium and phosphorus [5,22,25].

These findings suggest that more advanced statistical methods are required to adequately quantify the true relationship between these biochemical abnormalities and mortality risk in ESRD patients. Researchers have categorized markers (eg, quintiles, high/normal/low groups) as a way to account for non-linear relationships. A few have more rigorously used spline terms [20,21,26] (ie, mathematical functions used to model non-linear relationships) and/or tested for interactions [22,25] (effect modifiers). Unfortunately, since modeling non-linear relationships in regression models is complex, many studies have failed to do so, and the studies that have examined non-linear relationships between biochemical measures and mortality often have not provided results in a format amenable to a traditional meta-analysis.

Thus, the purpose of this study is two-fold. We will review the literature with a special emphasis on the statistical methods used to model the serum levels of PTH, phosphorus, and calcium with mortality. Next, we will use more statistically rigorous meta-analytic methods to improve the estimates of the relationships between biochemical markers of altered mineral metabolism and mortality among patients on dialysis.

## Methods

### Data sources and search strategy

We searched PubMed, Embase, and Cochrane for English-language articles published subsequent to Covic et al.’s review (ie, 2008–2010) using a search strategy that included terms related to the population, biochemical parameters, and outcomes of interest (see Additional file 1). We reviewed the studies on all-cause mortality among patients on dialysis that were cited by Covic et al. [17] and Palmer et al. [18]. We also reviewed reference lists of relevant primary research and review articles for additional publications.

### Initial inclusion criteria and data abstraction

To be included in this review, studies had to (1) have a patient population comprised of adults receiving hemodialysis or peritoneal dialysis; (2) report data on at least one of the selected biochemical parameters, namely calcium, phosphorus, and PTH; and (3) report findings for all-cause mortality. We excluded studies of patients not on dialysis (eg, pre-dialysis CKD, status post-renal transplant) as well as studies that only reported data on calcium-phosphorus product.

Relevant data from each study were abstracted into an evidence table by one investigator (JLN) and were verified by the study statistician (BHN) prior to use in the meta-analysis. Data elements included study characteristics (eg, study design, analytic approach, duration of follow-up), patient characteristics (eg, dialysis type and vintage, age, gender), biochemical parameters and units, time of measurement of biochemical parameters (eg, baseline, time-averaged), and mortality/survivor data.

### Literature assessment

For each manuscript that presented a correlation of all-cause mortality data with the biochemical measures of interest, we noted if the authors modeled the biochemical parameters as linear (ie, continuous) predictors of mortality or if they allowed a non-linear relationship with mortality (eg, modeled with splines, modeled as a categorical or binary variable). We also noted if figures were plotted showing the relationship between the parameters and mortality.

### Primary meta-analysis approach

To better approximate possible non-linear relationships between biochemical parameters and mortality, we assumed a piecewise linear relationship between each parameter and mortality, and we used each publication’s study-defined referent category. We then performed 2 separate meta-analyses to compare (1) values higher than the referent category vs referent, and (2) values lower than the referent category vs referent.

To be included in the quantitative meta-analysis, each study had to report data on PTH, calcium, and/or phosphorus as categorical variables with the following requirements: (1) the ranges of each category must be specified, (2) the referent category in the study’s multivariate analysis must be the “middle” (ie, both higher-than-referent and lower-than-referent categories were available for comparison), (3) the sample size of each category must be given, and (4) the number of deaths of patients in the multivariate analysis must be given. We also excluded studies with inadequate multivariate analysis (eg, only crude unadjusted results provided, only 1–2 covariates). When RRs were presented graphically without explicit quantitative details, we used a “pencil and ruler” method to extrapolate these values. We did not attempt to contact study authors to inquire about missing data or exact values of graphical data elements.

We also attempted to capture the following statistics for each study: (1) number of deaths per category, (2) person-years per category, (3) covariates in multivariate models, and (4) overall mean and SD of the biochemical parameter and the mean and SD at each categorical level. Unfortunately, both the number of deaths and person-years per biochemical parameter category were not reported in the studies that met our inclusion criteria, and the other statistics were often missing as well.

For the quantitative analysis, we employed the meta-analytic method proposed by Greenland and Longnecker [27] to handle results presented as categorically (with a referent level) and subsequently adapted and expanded upon by Hamling et al. [28]. The Hamling method allows for alternative comparisons of categorical data and is useful when the threshold values in each category are not uniform across studies. For example, studies of cigarette smoking exposure and cancer may present results based on number of pack-years of exposure, and those categories vary across studies. By applying Hamling’s method, one can group dissimilar categories to permit more consistent comparisons across disparate studies (eg, a meta-analysis of *any* smoking exposure vs. no smoking exposure). In our context, these were relatively high (or low) biochemical parameter values compared to referent.

We used the method of Hamling et al. [28] to derive 2 adjusted RR estimates for each study, one for high values versus the reference range and another for low values versus the reference range. These results were derived from the hazard ratios (HRs) given at the various categorical levels in each study after a multivariate Cox-regression was performed. We assumed a piecewise linear relationship between mortality and the biochemical parameter levels (categories) and that a correlated (non-zero) covariance exists among the series of log RRs. As such, we estimated a variance-covariance matrix of the beta coefficients using the meta-analytic method proposed by Greenland and Longnecker [27]. We also created a meta-regression with the dose estimate at the study level and the time to follow up at the study level as the independent covariate.

All meta-analyses were done with Stata 11.1, StataCorp LP, College Station, TX. Results from the Hamling method were derived from an Excel macro available at http://www.pnlee.co.uk/software.htm[28].

### Sensitivity analyses

Since the Hamling method requires an estimate of the number of deaths in the referent category and this was unknown, we conducted a sensitivity analysis by varying our initial mortality estimates in 2 ways: (1) 10% and 15% higher number of deaths, and (2) 10% and 15% lower number of deaths. These cutoffs were selected based on an educated assessment of our uncertainty regarding the number of deaths in the referent category. We then regenerated the meta-analysis results and examined how these variations affected the RRs.

### Simulation modeling

As an exploratory analysis, we simulated patient-level results from 5 observational studies selected for large sample size (Block et al. [10], Floege et al. [16], Kalantar-Zadeh et al. [14], Slinin et al. [5], and Tentori et al. [15]). Data for the 3 biochemical parameters were simulated for each of 100,000 hypothetical patients using a lognormal distribution calibrated from the published data of the 5 studies (eg, frequency distribution, mean, standard deviation, correlations between parameters) in order to optimally account for differences in categorization of the biochemical parameters by the respective observational studies. A non-linear dose-effect function (with functional form based on Floege et al. [16]) was applied to the simulated data and calibrated from the observed results. Simulation models were performed using Python 2.6.6 with NumPy 1.5.1rc2 and SciPy 0.8.0.

## Results

### Overview of studies

**Figure 2:**
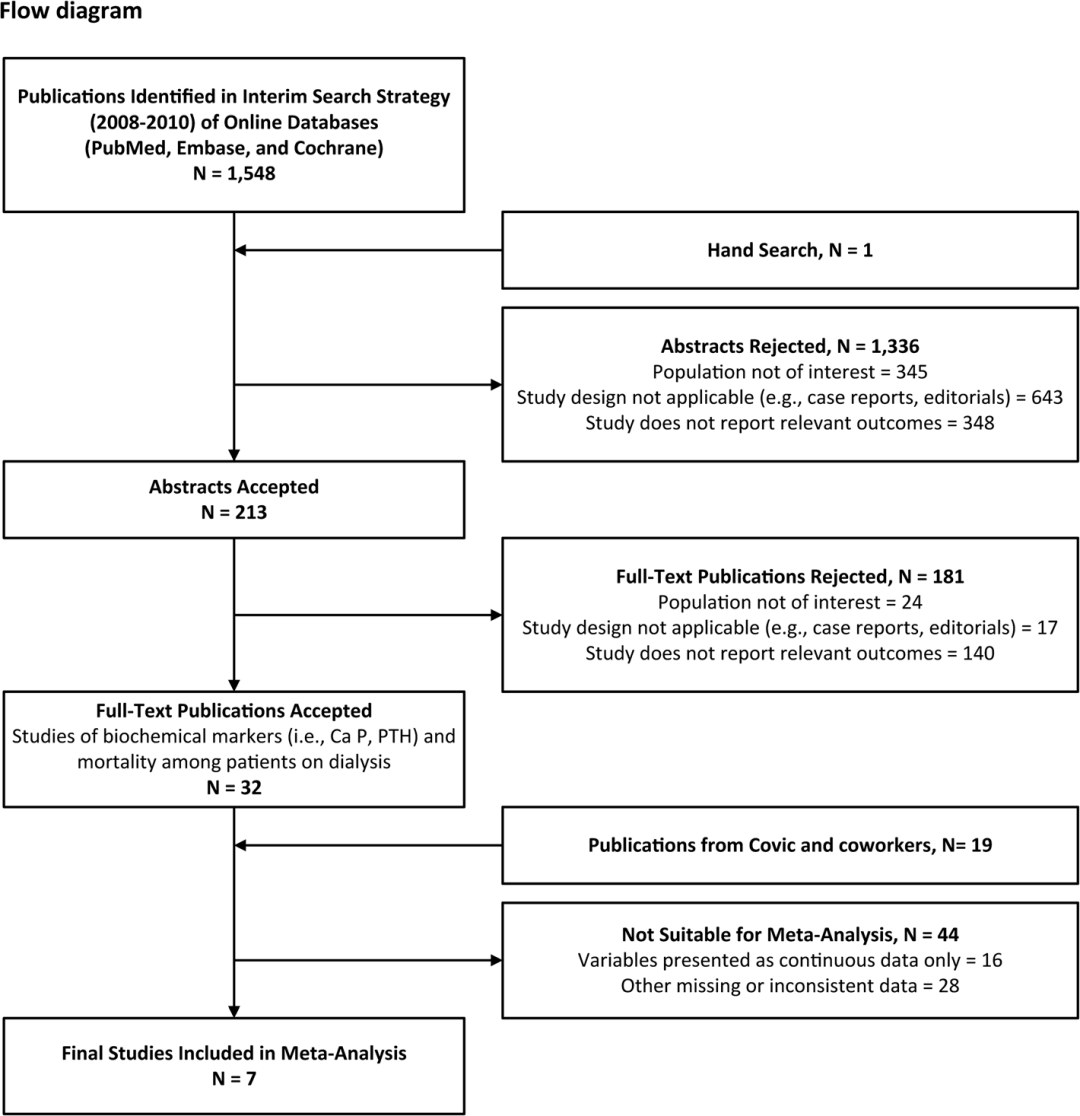
Flow diagram: publications documenting the association between relatively high/low biochemical parameters and mortality.

The interim search strategy identified 1,548 publications. We ultimately identified 51 studies among patients on dialysis that presented all-cause mortality data correlated with the biochemical measures of interest (see Figure 2 and Table 1).

**Table 1:** Overview of accepted publications

**Author, year**	**Study or database name**	**Study design**	**Duration of follow-up**	**Dialysis vintage**	**Dialysis type**	**Sample size**	Allowed for non-linear relationship?*	**Was the biochemical parameter associated with mortality after multivariate adjustment?**			**Graphical depiction?**^**†**^
								**PTH**	**Phosphorus**	**Calcium**	
***Studies with adequate data for meta-analysis***											
Block 2004 [10]	Fresenius Medical Care	Retrospective cohort	12-18 months (total)	Maintenance	HD	40,538	Yes	Yes	Yes	Yes	Yes (U-shaped for calcium, linear for albumin-adjusted calcium)
Dukkipati 2010 [29]	DaVita-NIED	Prospective cohort	63 months (total)	Maintenance	HD	748	Yes (for PTH only)	Yes	Not stated	Not stated	No
Floege 2011 [16]	European Fresenius Medical Care	Retrospective cohort	20.9 months (median)	Incident or Maintenance	HD	7,970	Yes (also used fractional polynomials)	Yes	Yes	Yes	Yes
Kalantar-Zadeh 2006 [14]	DaVita	Prospective cohort	24 months (total)	Maintenance	HD	58,058	Yes	Yes	Yes	Yes	Yes
Rodriguez-Benot 2005 [24]	Cordoba, Spain	Prospective cohort	97.6 months (median)	Maintenance	HD	385	Yes (for phosphorus and PTH only)	No	Yes	Yes	No
Tangri 2011 [30]	UKRR	Prospective cohort	24 months (total)	Incident	HD, PD	7,076	Yes	Yes	Yes	Yes	No
Tentori 2008 [15]	Dialysis Clinic Inc.	Prospective cohort	16.4 months (median)	Maintenance	HD	25,588	Yes	Yes	Yes	Yes	Yes
***Studies that did not qualify for meta-analysis***											
Block 1998 [31]	USRDS (CMAS and DMMS-1)	Retrospective cohort	2 years	Maintenance	HD	6,407	Yes (also analyzed as continuous linear predictor)	No	Yes	Yes (text and figure are inconsistent)	Yes (J-shaped for phosphorus, linear for calcium and PTH)
Block 2010 [32]	DaVita, CMS-ESRD	Prospective cohort	2.2 years	Maintenance	HD	25,150	Yes	Yes	Yes	Yes	No
Chang 2006 [33]	VA Cooperative Study #440	Post-hoc RCT	4 years	Incident or Maintenance	HD	197	Yes	No	Not measured	Not measured	No
Dreschler 2011 [34]	NECOSAD	Prospective cohort	6 years	Incident	HD, PD	1,628	Yes (for PTH only)	Yes	Not stated	Not stated	No
Dreschler 2011 [35]	NECOSAD	Prospective cohort	3 years	Incident	HD, PD	762	No	Not stated	Not stated	Not stated	No
Etter 2010[36]	*monitor!*	Prospective cohort	562 days (median)	Maintenance	HD	170	No	No	Not measured	Not measured	No
Foley 1996 [37]	Royal Victoria Hospital	Prospective cohort	5 years	Incident	HD, PD	433	Yes (calcium and phosphorus were binary)	Not measured	No	Yes	No
Ganesh 2001 [7]	USRDS (CMAS and DMMS 1/3/4)	Retrospective cohort	2 years	Maintenance	HD	12,833	Yes	Yes	Yes	Not stated	Yes (U-shaped for PTH)
Gutierrez 2008 [38]	ArMORR (Fresenius)	Prospective cohort	1 year	Incident	HD	10,044	Yes (for phosphorus only)	Not stated	Yes	Not stated	No
Hakemi 2010 [39]	Tehran, Iran	Prospective cohort	18.4 months (mean)	Maintenance	PD	282	Yes (for calcium and PTH only, binary)	Yes	Not measured	Yes	No
Hsiao 2011 [40]	Chang Gung Memorial Hospital	Prospective cohort	1 year	Maintenance	HD	109	No	No	No	Yes	No
Isakova 2009 [41]	ArMORR (Fresenius)	Prospective cohort	1 year	Incident	HD	10,044	Yes (for phosphorus only)	Not stated	Yes	Not stated	No
Jassal 1996 [42]	Belfast City Hospital	Prospective cohort	1 year	Incident	NR	99	No	Not measured	Yes (but not on older subset of patients)	Not measured	No
Jean 2009 [43]	Tassin la Demi-lune, France	Prospective cohort	2 years	Maintenance	HD	219	Yes (for phosphorus only, Not stated for PTH and calcium)	Not stated	No	Not stated	No
Kalantar-Zadeh 2010 [44]	DaVita-Los Angeles	Prospective cohort	5 years	Incident or Maintenance	HD	139,328	Yes	Yes	Yes	Yes	Yes (except for PTH)
Kimata 2007 [45]	DOPPS 1/2 (Japan)	Retrospective cohort	8,056 patient-years	Maintenance	HD	3,973	Yes (also analyzed as continuous linear predictors)	Yes, when linear No, when categorical	Yes, when categorical No, when linear	Yes, for both categorical and linear	Yes for phosphorus No for calcium or PTH
Komaba 2008 [46]	Takasago, Japan	Retrospective cohort	45 months	Maintenance	HD	99	Yes (binary only)	No	Yes (but analyzed with calcium only)	Yes (but analyzed with phosphorus only)	No
Lacson 2009 [47]	Fresenius Knowledge Center	Retrospective cohort	1 year	Maintenance	HD	78,420	In some models	Yes	Yes	Yes	Yes
Leggat 1998 [48]	USRDS (CMAS and DMMS)	Retrospective cohort	2 years	Maintenance	HD	6,251	Yes	Not measured	Yes	Not measured	No
Lin 2010 [49]	Chang Gung Memorial Hospital	Prospective cohort	18 months	Maintenance	PD	315	No	No	No	No	No
Lowrie 1990 [50]	National Medical Care	Cross-sectional	18 months	Maintenance	HD	19,746	No	Not measured	Yes	No	No
Lukowski 2010 [51]	DaVita	Retrospective cohort	3 years	Maintenance	HD	58,917	Yes (for phosphorus only, Not stated for PTH and calcium)	Not stated	Yes	Not stated	Yes for phosphorus No for calcium or PTH
Maeda 2009 [52]	Kyushu University Hospital	Prospective cohort	5 years	Maintenance	HD	226	No	No	No	No	No
McNeill 2010 [53]	DaVita	Cohort, unspecified	3 years	Maintenance	HD	58,917	Yes (for PTH only, Not stated for phosphorus and calcium)	Yes	Not stated	Not stated	Yes for PTH No for calcium or phosphorus
Melamed 2006 [54]	CHOICE	Prospective cohort	2.5 years (median)	Incident	HD, PD	1,007	Yes	Yes (as a time-dependent predictor)	Yes (as a time-dependent predictor)	Yes (as a time-dependent predictor)	No
Miller 2010 [22]	DaVita-Los Angeles	Retrospective cohort	5 years	Maintenance	HD	107,200	Yes	Yes	Yes	Yes	Yes for calcium No for phosphorus or PTH
Miller 2009 [55]	DaVita	Cohort, unspecified	5 years	Maintenance	HD	151,555	Yes	Yes	Yes	Yes	No
Morrone 2009 [56]	MMTE (Italy)	Prospective cohort	18.8 months (mean)	Incident	HD	411	Yes	Yes	Not stated	Not stated	Yes for PTH No for phosphorus or calcium
Naves-Diaz 2011 [57]	Fresenius-CORES	Retrospective cohort	4.5 years	Incident or Maintenance	HD	16,173	Yes	Yes	Yes	Yes	Yes
Naves-Diaz 2008 [58]	Fresenius-CORES	Retrospective cohort	4.5 years	Incident or Maintenance	HD	16,004	Yes	Yes	Yes	Yes	No
Noordzij 2008 [26]	NECOSAD	Prospective cohort	7.8 years	Incident	HD, PD	1,621	Yes (restricted cubic splines)	Not stated	Yes	Yes	Yes (spline)
Noordzij 2005 [23]	NECOSAD	Prospective cohort	7.5 years (max)	Incident	HD, PD	1,629	Yes	No	Yes	No	No
Phelan 2008 [59]	Beaumon Hospital (Ireland)	Retrospective cohort	4.5 years (mean)	Incident	HD, PD	1,007	Yes (binary only)	No	Yes	No	No
Port 2004 [60]	DOPPS I	Prospective cohort	5 years	Maintenance	HD	17,245	Yes (for phosphorus only, binary)	Not measured	Yes	Not measured	No
Saran 2003 [61]	DOPPS	Retrospective cohort	1.8-2.9 years (median, depending on region)	Maintenance	HD	14,930	Yes (for phosphorus only, binary)	No	Yes	Not measured	No
Shinaberger 2008 [20]	DaVita	Retrospective cohort	3 years	Maintenance	HD	30,075	Yes (restricted cubic splines)	Not stated	Yes	Not stated	Yes
Shinaberger 2008 [21]	DaVita	Retrospective cohort	3 years	Maintenance	HD	34,307	Yes (restricted cubic splines, PTH only)	Yes	Not stated	Not stated	Yes
Simic-Ogrizovic 2009 [62]	Clinical Center of Serbia	Prospective cohort	3 years	Maintenance	HD	130	No	Not measured	Yes	No	No
Sit 2008 [63]	Dicle University	Retrospective cohort	10 years	Maintenance	NR	1,538	No	Yes	No	No	No
Slinin 2005 [5]	USRDS (1/2/3)	Retrospective cohort	3.9 years (mean)	Maintenance	HD	14,829	Yes	Yes	Yes	Yes	No
Stevens 2004 [25]	PROMIS	Prospective cohort	2.6 years (median)	Maintenance	HD, PD	515	Yes (examined interactions; also modeled continuous, linear predictors)	No, when continuous Yes, when categorical with interactions	Yes, for both continuous and categorical with interactions	No, when continuous Yes, when categorical with interactions	No
Takemoto 2009 [64]	Toranomon Hospital	Prospective cohort	8 years	Maintenance	HD	68	No	Yes	No	No	No
Wald 2008 [65]	HEMO	Post-hoc RCT	6.5 years	Maintenance	HD	1,846	Yes	No	Yes	Yes	Yes for calcium and phosphorus No for PTH
Young 2004 [66]	DOPPS 1/2	Retrospective cohort	2 years	Maintenance	HD	15,475	No	Yes	Yes	Yes	No

HD, hemodialysis; NR, not reported; PD, peritoneal dialysis; PTH, parathyroid hormone; RCT, randomized controlled trial.

* Study explicitly allowed for a non-linear relationship between biochemical parameters and mortality (eg, use of a categorical variable or splines).

†Study provided a graphical depiction of the relationship between biochemical parameters and mortality.

As noted in Table 1, most studies found a significant relationship between the 3 biochemical measures and mortality. Twelve studies (23.5%) modeled the biochemical measures as linear, continuous variables. Furthermore, no paper included explicit discussion of testing for non-linearity to justify this decision. Another 14 studies (27.5%) attempted to model these variables in a non-linear manner but only did so for some measures (eg, just phosphorus) or merely dichotomized the variable rather than use multiple categories or splines. Only 5 of 51 studies used spline functions even though this is the most rigorous method to handle non-linearity and prevents a loss of power compared to categorization [67].

The choice of a linear or non-linear model also affected results. In 8 studies that measured PTH and modeled it linearly, 4 (50.0%) found no significant association with mortality. In contrast, in the 37 studies that modeled PTH with categories or splines, just 8 (21.6%) found no significant association. For phosphorus, 5 of 11 studies with linear models (45.5%) found no association with mortality compared to 2 of 37 studies (5.4%) in which phosphorus was modeled as a non-linear predictor. Similarly for calcium, 4 of 10 studies with linear models (40.0%) explicitly stated no association with mortality compared to 2 of 37 non-linear studies (5.4%). Nineteen of 51 studies graphed at least one categorized predictor (eg, by quartile) by either percent dying or by adjusted RR. In 18 of 19 studies (94.7%), a non-linear U or J shape was evident for at least one of these variables.

### Primary meta-analysis findings

Seven studies were included in the meta-analysis (see Table 2) because the majority of candidate reports did not contain the necessary data elements. Of the 7 studies, 4 included relevant data on phosphorus [10,14,24,30], 3 on calcium [10,14,30], and 5 on PTH [14-16,29,30]. Although we made no restrictions based on study design or dialysis type, all of the included studies were prospective or retrospective cohort studies, and virtually all patients received hemodialysis.

**Table 2:** Summary of studies included in meta-analysis

**Author, year**	**Study or database name**	**Study design**	**Duration of follow-up**	**Dialysis vintage**	**Dialysis type**	**Sample size**	**Biochemical parameters with adequate data**	**Timing of assessment**	**Model adjustment**
Block, 2004 [10]	Fresenius Medical Care	Retrospective cohort	12-18 months (total)	Maintenance	HD	40,538	P, Ca	Average of values in first 3 months of study	Multivariate adjusted
Dukkipati, 2010 [29]	DaVita-NIED	Prospective cohort	63 months (total)	Maintenance	HD	748	PTH	Baseline, drawn prior to dialysis session	Multivariate (MICS) adjusted
Floege, 2011 [16]	European Fresenius Medical Care	Retrospective cohort	20.9 months (median)	Incident, 35% Maintenance, 65%	HD	7,970	PTH	Average of values in 1st quarter of follow-up	Multivariate, time-dependent adjusted
Kalantar-Zadeh, 2006 [14]	DaVita	Prospective cohort	24 months (total)	Maintenance	HD	58,058	P, Ca, PTH	Average of values in 1st quarter of follow-up	Multivariate (MICS), time-dependent adjusted
Rodriguez-Benot, 2005 [24]	Cordoba, Spain	Prospective cohort	97.6 months (median)	Maintenance	HD	385	P	6-month mean prior to death or end of study period	Multivariate adjusted
Tangri, 2011 [30]	UKRR	Prospective cohort	24 months (total)	Incident	HD, 70% PD, 30%	7,076	P, Ca, PTH	Average of values during the first year of dialysis	Multivariate adjusted
Tentori, 2008 [15]	Dialysis Clinic Inc.	Prospective cohort	16.4 months (median)	Maintenance	HD	25,588	PTH*	Baseline	Multivariate adjusted

Ca, calcium; HD, hemodialysis; MICS, malnutrition-inflammation complex syndrome; NIED, Nutritional and Inflammatory Evaluation in Dialysis; P, phosphorus; PD, peritoneal dialysis; PTH, parathyroid hormone; UKRR, United Kingdom Renal Registry.

*Excluded data for the other biochemical parameters due to discrepancies between sample sizes presented in publication tables and manuscript text.

**Figure 3:**
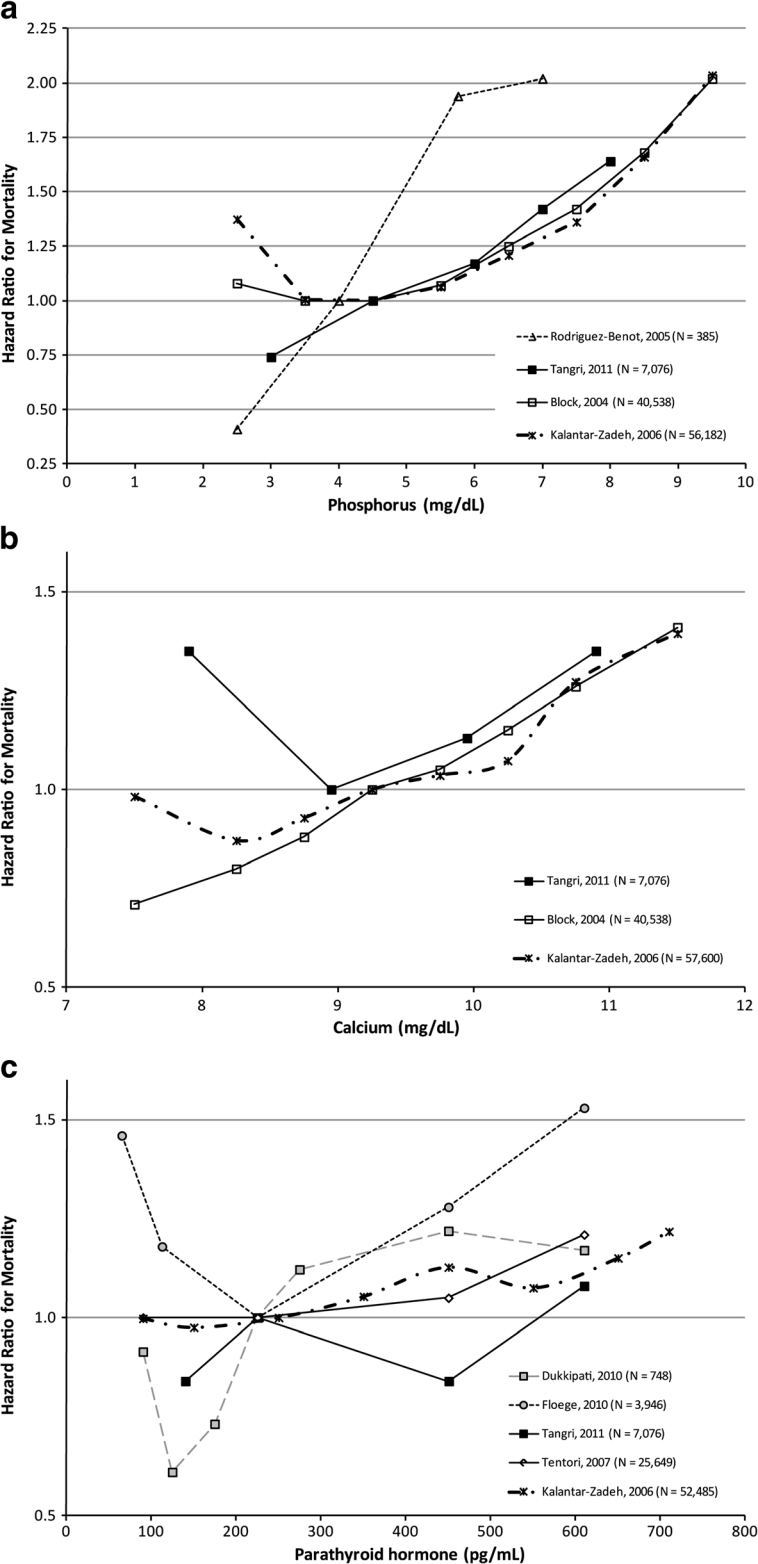
**Studies used in the meta-analysis of biochemical parameters and mortality. ***In these studies, biochemical parameter levels were analyzed as categorical variables. In order to visualize the relationship between mortality and the biochemical parameters, the values on the x-axis for each symbol in the figure represent the midpoint within each category for each study. In order to make the graphical representation more comparable across studies, we adjusted the HRs so that the midpoint of the reference range was as similar as possible. We stress that this is done just for the figures only and not for the meta-analyses. Specific adjustments are noted below.***a**. Phosphorus: The midpoints of reference ranges were ≈4- 4.5 mg/dL across all studies with the exception of Kalantar-Zadeh 2006 [14]. For this study, we adjusted the graphical depiction of the reference range from 5–6 mg/dL to 4–5 mg/dL. Of note, non-linear relationships were most clearly evident in the 2 studies with the largest sample sizes. **b**. Calcium: The midpoints of reference ranges were ≈9-9.25 mg/dL across all studies. No studies had to be adjusted for graphical display. **c**. PTH: The midpoints of reference ranges were ≈225-250 pg/mL across all studies with the exception of Dukkipati 2010 [29]. For this study, we adjusted the graphical depiction of the reference range from 300–600 pg/mL to 200–249 pg/mL.

Reference ranges were generally consistent across studies. The midpoints of the reference ranges were ≈4-4.5 mg/dL for phosphorus (with the exception of Kalantar-Zadeh 2006 [14], which was 5.5 mg/dL), ≈9-9.25 mg/dL for calcium, and ≈225-250 pg/mL for PTH (with the exception of Dukkipati 2010 [29], which was 450 pg/mL). Figures 3a-c provide graphical depictions of the HRs from the studies used in each meta-analysis.

We present the adjusted RRs based on the Hamling method for each study in Table 3 (relatively high values versus referent) and Table 4 (relatively low versus referent). Table 5 summarizes the derived RRs from each meta-analysis. Higher-than-referent levels of phosphorus (RR = 1.20, 95% CI=1.15-1.25), calcium (RR = 1.10, 95% CI=1.05-1.14), and PTH (RR = 1.11, 95% CI=1.07-1.16) were all significantly associated with an increased risk of mortality. Although no significant associations between relatively low phosphorus or PTH and mortality were observed, there did appear to be a protective effect for relatively low calcium and mortality (RR = 0.86, 95% CI = 0.83-0.89).

**Table 3:** Mortality risk for relatively high biochemical parameter values versus referent values

**Study**	**Reference range**	N*	**RR for high values vs. referent (95% CI)**	**Follow-up (months)**^**†**^
***Phosphorus (mg/dL)***				
Block 2004 [10]	4.0-5.0	35,783	1.21 (1.14-1.29)	18
Kalantar-Zadeh 2006 [14]	5.0-6.0	36,955	1.20 (1.13-1.29)	24
Rodriguez-Benot 2005 [24]	3.0-5.0	375	1.96 (1.20-3.18)	97.6
Tangri 2010 [30]	3.5-5.5	6,538	0.74 (0.53-1.03)	24
***Calcium (mg/dL)***				
Block 2004 [10]	9.0-9.5	23,168	1.14 (1.06-1.22)	30
Kalantar-Zadeh 2006 [14]	9.0-9.5	36,831	1.06 (1.00-1.12)	24
Tangri 2010 [30]	8.4-9.5	6,843	1.23 (0.95-1.58)	24
***Parathyroid hormone (pg/mL)***				
Dukkipati 2010 [29]	300-600	288	0.96 (0.60-1.55)	63
Floege 2010 [16]	150-300	2,443	1.35 (1.14-1.61)	20.9
Kalantar-Zadeh 2006 [14]	200-300	29,273	1.12 (1.06-1.18)	24
Tangri 2010 [30]	151-300	4,842	0.92 (0.71-1.20)	24
Tentori 2008 [15]	100-300	18,224	1.09 (1.02-1.16)	18.7

CI, confidence interval; RR, risk ratio.

*Sample size includes patients in the reference range plus those with relatively high biochemical parameter values.

†The median or mean (or total, if median was not given) follow-up time in each study.

**Table 4:** Mortality risk for relatively low biochemical parameter values versus referent values

**Study**	**Reference range**	N*	**RR for low values vs. referent (95% CI)**	**Follow-up (months)**^**†**^
***Phosphorus (mg/dL)***				
Block 2004 [10]	4.0-5.0	13,478	1.01 (0.95-1.08)	18
Kalantar-Zadeh 2006 [14]	5.0-6.0	34,636	0.99 (0.88-1.12)	24
Rodriguez-Benot 2005 [24]	3.0-5.0	170	0.41 (0.05-3.26)	97.6
Tangri 2011 [30]	3.5-5.5	4,776	1.22 (1.01-1.49)	24
***Calcium (mg/dL)***				
Block 2004 [10]	9.0-9.5	28,129	0.81 (0.77-0.85)	30
Kalantar-Zadeh 2006 [14]	9.0-9.5	37,662	0.93 (0.87-0.98)	24
Tangri 2011 [30]	8.4-9.5	3,394	1.35 (0.24-7.48)	24
***Parathyroid hormone (pg/mL)***				
Dukkipati 2010 [29]	300-600	648	0.72 (0.52-0.95)	63
Floege 2010 [16]	150-300	2,595	1.31 (1.14-1.51)	20.9
Kalantar-Zadeh 2006 [14]	200-300	33,758	0.99 (0.94-1.05)	24
Tangri 2011 [30]	151-300	5,071	0.84 (0.61-1.16)	24
Tentori 2008 [15]	100-300	17,648	1.00 (0.93-1.08)	18.7

CI, confidence interval; RR, risk ratio.

*Sample size includes patients in the reference range plus those with relatively low biochemical parameter values.

†The median or mean (or total, if median was not given) follow-up time in each study.

**Table 5:** Summary of findings: meta-analyses of mortality risk for relatively high/low biochemical parameter values versus referent values*

**Biochemical parameter**	**Mortality risk for relatively low biochemical parameter values**		**Mortality risk for relatively high biochemical parameter values**	
	**Risk ratio (95% CI)**	**I**^**2**^	**Risk ratio (95% CI)**	**I**^**2**^
**Phosphorus**	1.02 (0.97-1.08)	30.5%	1.20 (1.15-1.25)	75.2%
**Calcium**	0.86 (0.83-0.89)	84.0%	1.10 (1.05-1.14)	39.9%
**PTH**	1.01 (0.97-1.05)	79.8%	1.11 (1.07-1.16)	47.8%

*Statistical heterogeneity: the I^2^ statistic describes the percent of variation in the risk ratio that is attributable to heterogeneity in a meta-analysis.

We conducted 6 meta-regressions to examine the effect of follow-up time on the RR estimates, one for each biochemical parameter at relatively low and high values. Results from the meta-regression indicated that follow-up time did not have a statistically significant effect on the RRs (data not shown).

### Sensitivity analysis

We varied the baseline estimated number of deaths and non-deaths in the referent group when doing the Hamling analysis by 10% to 15% in both directions. Results of these sensitivity analyses demonstrated that the estimated RR values were extremely stable and almost identical to the original estimates (data not shown). Due to the dissimilar reference range used by Dukkipati, we also re-ran the original meta-analyses without this study. The overall effect estimates and measures of heterogeneity were not appreciably different from those in the primary analysis (data not shown).

### Simulation analysis

Preliminary work on a simulation of 5 large observational studies (Block et al. [10], Floege et al. [16], Kalantar-Zadeh et al. [14], Slinin et al. [5], and Tentori et al. [15]), in which we estimated continuous functions for the relationship between mortality and each of the 3 biochemical parameters also showed non-linear U- or J-shaped trends that were a good fit with the published data. Similar to the meta-analysis, the simulation showed significant heterogeneity among the studies for all 3 biochemical parameters. The source of the heterogeneity was not obvious.

## Discussion

Our literature review shows that most studies found a significant relationship between serum levels of PTH, calcium, and phosphorus and mortality even though a sizeable number naively modeled these variables as linear predictors. Another large group of studies only examined these variables as binary variables, which is a methodologically weak approach. For example, Phelan et al. [59] found no relationship between PTH above and below 300 pg/ml and mortality. Interestingly, they correctly deduced that this result may be because very high PTH values could have “cancelled out” very low PTH values and both extremes may be harmful. However, they did not take corrective actions (eg, more categories, spline functions) in their methods. We also rarely encountered a paper that reported explicit power calculations, which is important given that categorizing a variable reduces power. Thus, it is important to acknowledge that the statistical methods used in the literature may be insufficient and the results should be cautiously interpreted.

In our meta-analysis, higher-than-referent (often “normal”) serum levels of PTH, calcium, and phosphorus in dialysis patients were positively associated with increased mortality. For lower-than-referent values, findings were less consistent across the 3 selected parameters; we observed no significant association for phosphorus and PTH and reduced mortality for relatively low values of calcium. The correlation between increased mortality and elevated biochemical parameter values was expected based on the assumption that levels within a “normal” range represent optimal health. The lack of statistical significance for lower values of phosphorus and PTH may be partly attributable to the small numbers of dialysis patients with sub-referent levels, leading to low statistical power. Although low serum calcium is not typically considered “healthy,” the association with significantly lower mortality risk is not completely surprising and may be explained by lower vascular calcification and associated mortality [10,68-72].

Although our findings for phosphorus and mortality are in line with those of the recent meta-analysis by Palmer et al. [18], our findings for calcium and PTH differ as Palmer et al. did not demonstrate a significant mortality risk for either calcium or PTH. There are several reasons for this disparity. First, the Palmer study assumed a linear relationship between the biochemical parameters and mortality over the total range of values, while our study used a piecewise linear approach that allowed for the possibility of U-shaped or J-shaped relationships. As Figure 1 illustrates, if the relationship is U-shaped or J-shaped, a linear assumption may incorrectly estimate the magnitude of the association. In fact, when studies did model a biochemical parameter as both a continuous, linear predictor and then as a categorical variable, the RRs were more extreme with the categorical approach [45,47]. The Palmer study also included studies of patients who were not on dialysis, such as patients with pre-dialysis CKD and ESRD patients who had received kidney transplants. If the relationship between abnormal biochemical parameters and mortality is strongest among the sickest patients (ie, those on dialysis), this would further attenuate the estimated relationship. Furthermore, some studies that were included in Palmer’s meta-analysis could not be utilized with our meta-analytic approach as our requirements for analysis were more stringent.

Using very similar review methods to ours, Covic et al. [17] concluded that quantitative synthesis in a meta-analysis was not possible due to significant clinical and methodological heterogeneity across the identified studies. Although we attempted to resolve this by including additional, more recent studies and by focusing on a more clinically narrow population of ESRD patients, we still observed clinical and methodological heterogeneity in the included studies. Aside from the types of differences documented by Covic et al. [17], including variations for dialysis vintage and covariates included in the respective multivariate models, we theorize that the clinical context of these studies may be an important source of heterogeneity. For example, management of biochemical abnormalities and/or other aspects of patient care may vary across institutions or according to geographic region (eg, vitamin D use). It is worth noting that most studies found a significant association with all 3 variables and mortality and when the variables were graphed against mortality, frequently a U or J shape was observed.

Our meta-analysis has specific limitations. Although we did not perform independent, dual data abstraction (the gold standard for systematic reviews), we employed a rigorous quality review of abstracted data, and we do not believe that this approach compromised the accuracy of our results. Additionally, while several statistically significant associations between biochemical parameters and mortality were found, we stress that association alone does not necessitate a causal relationship. For example, relatively high values of any of the 3 biochemical parameters may reflect the general health of a patient, which may not change as a result of treatment geared to normalizing a single abnormality. Furthermore, many patients in these observational studies received medications aimed at correcting (or preventing) out-of-range levels; their ability to respond to such therapies may have reflected their general health.

In addition, we excluded several large studies because their results could not be synthesized in our meta-analysis due to the way the results were reported. All meta-analyses are limited by the availability of pertinent data in relevant publications, and the study by Palmer et al. also excluded many studies for a variety of reasons. Nevertheless, our analysis of abnormally low values may have been underpowered and we encourage future studies to look at this clinically uncommon subset of patients in more detail.

Our study analyzed the association between biochemical parameter values above and below the referent value of a study in order to better approximate non-linearity, although this approach still assumes linear relationships above and below the reference range. However, not all studies used a reference range that would be expected to include the nadir of the curve, which leads to a lower-than-expected slope estimate for the segment of the curve that includes the nadir of the U-shape or J-shape. This underestimation is similar to that due to assuming a linear effect, although the magnitude of bias is much smaller. The 2 studies [14,29] that chose a reference range outside of “normal” bounds both used values that were higher than the other studies with which they were meta-analyzed; this may have led to an underestimation of the effect of relatively low values.

Finally, the historical approach to altered mineral metabolism has been somewhat reductionist as studies tend to evaluate individual biochemical parameters in isolation and assume independence across those parameters. Unfortunately, a more comprehensive approach that explores the interactions between biochemical parameters for mortality effects has not been well researched [25]. While some studies reported data on calcium-phosphorus product, not all investigated the extent to which their results showed interaction between calcium and phosphorus, and the main effects of calcium and phosphorus were not always reported, which is a major methodological shortcoming. Therefore, our meta-analysis did not consider interactions. The lack of investigation of interactions further hinders direct applicability of the results of observational studies to determine the quantitative targets of practice guidelines.

## Conclusions

In summary, we conclude that the relationships between phosphorus, calcium, and PTH and mortality among ESRD patients receiving dialysis appear to not be linear but rather trend towards non-linear U-shaped or J-shaped curves. In addition, we found that elevated values of all 3 of these biochemical parameters were associated with increased mortality. Also, our literature review shows that many studies have naively modeled the relationship between these biochemical markers and mortality making their interpretation problematic at best. Prior meta-analyses of studies that have not correctly modeled these biochemical markers cannot be considered definitive. Instead, we suggest that future studies use more advanced modeling techniques (eg, spline terms, interactions) on large datasets in order to better describe possible non-linear relationships between the biochemical markers and mortality.

## Competing interests

This study was supported by Amgen Inc. Authors SC, WG, and VB are all employees and stockholders in Amgen Inc.

## Author’s contributions

JN was responsible for the design and conduct of the systematic review, design of meta-analysis, interpretation of results, and composition of all sections of the manuscript. RB was responsible for the design of the meta-analysis, interpretation of results, composition of the Discussion section, and review of the manuscript. BN was responsible for the design and conduct of the meta-analysis, interpretation of results, and composition of all sections of the manuscript. RM was responsible for the design of the systematic review, interpretation of results, and review of the manuscript. SC, WG, and VB are all employees and stockholders in Amgen Inc and each contributed to the interpretation of results, writing and review of the manuscript. All authors read and approved the final manuscript.

## Pre-publication history

The pre-publication history for this paper can be accessed here:

http://www.biomedcentral.com/1471-2369/14/88/prepub

## Supplementary Material

Additional file 1 **Search Strategy (adapted from Covic et al. conducted on December 5, 2010). ***Note: The search strategy was created in PubMed and adapted as necessary for Embase and Cochrane.* Click here for file
