# Supplementary material for: Is there an association between elevated or low serum levels of phosphorus, parathyroid hormone, and calcium and mortality in patients with end stage renal disease? A meta-analysis
Source: BMC Nephrol. 2013 Apr 17;14:88. doi: 10.1186/1471-2369-14-88 (PMC3658973; doi:10.1186/1471-2369-14-88)
Supplement: Additional file 1 — Search Strategy (adapted from Covic et al. conducted on December 5, 2010). Note: The search strategy was created in PubMed and adapted as necessary for Embase and Cochrane. [file 1471-2369-14-88-S1.doc]

**Additional file 1: Search Strategy (adapted from Covic et al; conducted on December 5, 2010)**

*Note: The search strategy was created in PubMed and adapted as necessary for Embase and Cochrane.*

| **Search** | **Category** | **Search Terms** |
| --- | --- | --- |
| 1 | Population | renal dialysis[mh] OR kidney failure, chronic[mh] OR kidney failure[mh] OR dialysis[mh] OR dialysis, renal OR hemodialysis OR peritoneal dialysis OR ESRD OR end-stage renal disease OR renal disease, end-stage OR renal failure, end-stage OR renal failure, chronic OR chronic kidney failure OR end-stage kidney disease OR chronic kidney disease OR chronic renal disease OR chronic renal failure OR renal failure OR dialysis |
| 2 | Biochemical parameters | parathyroid hormone[mh] OR calcium[mh] OR hypercalcaemia[mh] OR hypocalcaemia[mh] OR phosphorus[mh] OR parathyroid hormone OR PTH OR calcium OR hypercalcaemia OR hypocalcaemia OR serum calcium OR serum calcium level OR phosphorus OR serum phosphorus OR serum phosphorus level OR calcium-phosphorus product OR Ca x P product OR calcium x phosphorus product OR hyperphosphataemia |
| 3 | Interventions | calcitriol[mh] OR vitamin D[mh] OR calcium compounds[mh] OR calcium[mh] OR calcium carbonate[mh] OR cinacalcet OR cinacalcet hydrochloride OR Sensipar OR AMG073 OR AMG 073 OR Mimpara OR calcitriol OR alfacalcidol OR vitamin D OR vitamin D analogues OR paricalcitol OR Zemplar OR doxercalciferol OR Hectorol OR maxacalcitol OR falecalcitriol OR phosphate binders OR phosphate binder OR calcium acetate OR Phoslo OR calcium carbonate OR sevelamer hydrochloride OR sevelamer OR Renagel OR lanthanum carbonate OR Fosrenol OR ferric citrate OR calcium compounds OR calcium |
| 4 | Outcomes | mortality[mh] OR fatal outcome[mh] OR mortality OR overall mortality OR cardiovascular mortality OR death rate or case fatality rate OR death OR deaths OR myocardial infarction[mh] OR myocardial infarct OR cerebrovascular accident[mh] OR cerebral stroke OR cerebrovascular stroke OR stroke OR congestive heart failure OR cerebrovascular accident OR CVA OR acute coronary syndrome OR ACS OR transient ischaemic attack OR TIA |
| 6 | Combine | #1 AND #2 AND #4 |
| 7 | Combine | #1 AND #3 AND #4 |
| 8 | Combine | #6 OR #7 |
| 10 | Apply limits | Language: English  Added to Database: January 1, 2008 – December 5, 2010 |
